# Malarial Hæmoglobinuria

**Published:** 1892-04

**Authors:** H. E. Williams

**Affiliations:** Fordyce, Arkansas


### MALARIAL HÆMOGLOBINURIA.

BY H. E. WILLIAMS, M. D., FORDYCE, ARKANSAS.

I have been watching with particular interest numerous articles relative to hæmoglobinuria and its treatment, and appearing in different medical journals of recent date.

There seems to be much difference of opinion with physicians as to treatment, also as to the pathological condition that exists. I have had considerable experience with this fearful malady, having practiced medicine in the Mississippi swamps for ten years, and treating a great many cases each year, I have consequently seen it in every form, from the mildest to the severest.

As I have been solicited by some medical friends to offer for publication my plan of treatment, it will become necessary that I give my views as to the etiology and pathology in order to arrive at a correct basis for treatment.

We are all agreed that this disease is dependent upon malarial bacteria—those minute organisms being developed in a low marshy country, by high temperature, decomposition of vegetable matter, and stagnant water. These germs being developed, find their way into the human body by way of the digestive and respiratory organs, and the cutaneous surface; hence,as the disease is due to these germs, we must class it with the zymotic affections. The bacteria, after entering the circulation, produce more or less destruction of the blood plasma, and the fibrinogen of the red blood corpuscles (from zymosis) ; the red corpuscles, being set free in a state of disintegration, liberate its coloring matter (hæmoglobin). Now, as the red corpuscles are in a state of disintegration, they can no longer act as oxygen-bearers; consequently, this mass of broken down blood becomes effete matter. The coloring matter being set free, finds its way by general extravasation throughout the entire body. Nature now makes an effort to eliminate this effete matter by way of all the emunctories of the body; hence the bloody colored urine, rapid jaundice, black vomit. By way of digression, I will here state that the jaundiced condition is not (as was formerly supposed) due to extravasation of bile, but is due to diffusion throughout the body of this disintegrated blood, or coloring matter. As proof of this, I have often witnessed, after the application of a blister, a bloody-colored fluid in the vesicles, instead of the characteristic yellow fluid usually found in blisters.

Hæmoglobinuria attacks those who have been suffering with chronic malaria and chills. It is ushered in with a pronounced chill, followed by high fever, thirst and vomiting, rapid pulse, tongue usually coated with a heavy brown coat, headache, pain in lumbar region, rapid jaundice, bloody-colored urine; in the course of two or three hours a slight defervescence occurs, the skin begins to act, and the patient expresses himself as feeling better; but soon after a rigor occurs, with a rapid rise of temperature, with all the former symptoms more aggravated.

If left alone, these rigors occur about every four or five hours, each one rendering the case more grave, until finally black vomit, uræmic convulsions, or coma, with suppression of urine develops, and the case soon terminates fatally.

Now, how are we to treat a case like this? Medical men unacquainted with this formidable disease would very naturally suggest that, as this is one of the malarial affections, that we use quinine, it being considered a panacea in malaria; but from ten years' experience with this disease, I was forced to the conclusion that the use of it was not only a mistake, but a very grave one, I never having seen a case recover where quinine was used, even moderately.

There are certain conditions present in this disease that will not admit of the use of quinine, namely, high arterial pressure, deficient oxygenation of blood, due to destruction of red cells, defective elimination of urea, together with other products of waste, and a tendency to suppression of urine.

We are all aware that quinine, even in medium doses, diminishes the secretions of skin, gastro-intestinal and renal—the very conditions we don't want present. H. C. Wood's Materia Medica, edition of 1881, page 74, says: Ranke noticed that quiniæ lessened elimination of uric acid, also the other constituents of the urine. Zuntz found that twenty-five grains of quiniæ reduced his elimination of urea nearly 40 per cent.

Now, why should we persist in the use of quinine in this disease, when we see the effects are inimical to the conditions we have to combat? We augment the defective elimination of urea that already exists; dry up the renal secretion and hasten uremic coma, and I might add, kill our patient, for I do think a patient has hardly one chance in a thousand to recover if under the care of a physician using quinine.

Since abandoning quinine, my treatment has been to give an active mercurial course to arouse the alimentary tract.

℞ Hydrarg. chlo. mit., gr. xv.
Ipecac pulv., gr. iii.
M. ft. chartes No. 3.
Sig.: One powder every three hours.

As an antiseptic, I have used with excellent results, antipyrine, guarding the action of the heart with digitalis, if necessary, say—

℞ Antipyrine, ʒ ss.

Ft. chartes No. 5.

Sig.: One every three hours until fever subsides; after which I give eucalyptol in ten drop doses every three hours in capsules. I have found it to be a good antiperiodic, and a fine reconstructive agent, as well as a germicide.

From the beginning, I use sulphite of soda for its antizymotic action, and carry it to the point of saturation as early as possible.

I never meddle with the kidneys unless they begin to suspend action, or the flow so excessive as to threaten life by exhaustion. If the kidneys begin to fail, I use

℞ Tr. digitalis, ʒ iv.

Potass. nitr., ʒ iv.

Fl. ex. scoparin, ʒ vi.

Aqua, q. s., ℥ iii.

M. Sig.: Teaspoonful every 3 hours.

In addition to this, I usually flush the colon with warm water from time to time for its revulsive effect on the kidneys. I make no effort to check the so-called hæmorrhage, for I consider it an effort of nature to relieve itself of this effete matter, and should not be checked.

When convalescênce is established, the patient will need a tonic. I have found this to act nicely:

℞ Tr. ferri chlo. ʒ vi.

Acid phos. dil., ʒ iv.

Tr. nucis vom., ʒ v.

Aqua, q. s., ℥ iv.

M. Sig.: Teaspoonful three times daily, after meals.

Since adopting the above plan of treatment in this disease, I have lost very few cases, and all of them, when I used quinine

